# Supplementary material for: Identification of photoperiod-induced specific miRNAs in the adrenal glands of Sunite sheep (Ovis aries)
Source: Front Vet Sci. 2022 Jul 22;9:888207. doi: 10.3389/fvets.2022.888207 (PMC9354845; doi:10.3389/fvets.2022.888207)
Supplement: Supplementary file 1 [file Data_Sheet_1.ZIP › Supplementary file/miRDeep2 file.zip!/miRDeep2 file/Know_Structure/SPLP42A2/oar-mir-107.pdf]

miRBase precursor : oar-mir-107
Total read count : 5832
oar-miR-107 read count : 5827
remaining reads : 5

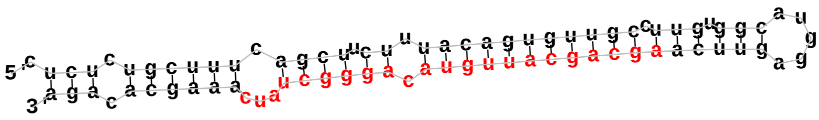

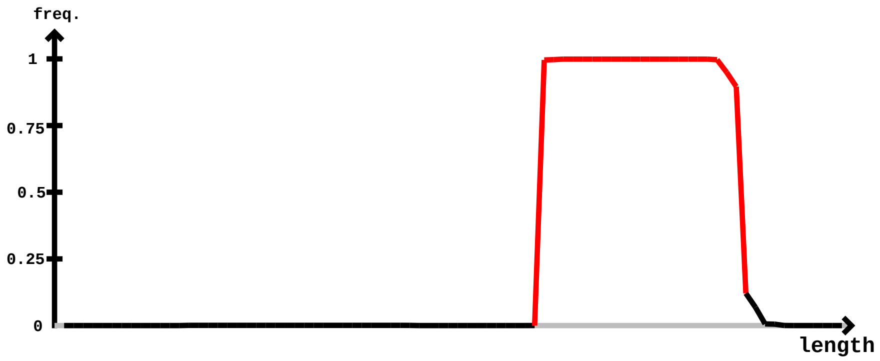

oar-miR-107

```
5'- cucucugcuuucagcuucuuuacaguguugccuuguggcauggaguucaagcagcauuguacagggcuaucaaagcacaga  -3'   exp
    .(((.((((((.((((.((.(((((((((((.(((.(((.....))))))))))))))))).))))))...)))))).)))   reads   mm   sample
    ............agcuucuuuacagugCugccuug..............................................   1       1    seq
    ............agcuucuuuacaguguugccuugu.............................................   2       0    seq
    ............agcuucuuuacagugCugccuugu.............................................   2       1    seq
    ................................................aagcagcauuguacagggcuau...........   1       0    seq
    .................................................agcagcauuguacaggg...............   1       0    seq
    .................................................agcagcauuguacagggc..............   12      0    seq
    .................................................agcagcauuguacagggcu.............   274     0    seq
    .................................................agcagcauuguacUgggcu.............   1       1    seq
    .................................................agcagcauuguacagggcua............   313     0    seq
    .................................................agcagcauuguacagggcuau...........   4508    0    seq
    .................................................agcagcauuguacagggcuauc..........   303     0    seq
    .................................................agcagcauuguacagggcuauca.........   362     0    seq
    .................................................agcagcauuguacagggcuaucaa........   5       0    seq
    .................................................agcagcauuguacagggcuauGaaa.......   26      1    seq
    .................................................agcagcauuguacagggcuauGaaag......   2       1    seq
    ..................................................gcagcauuguacagggcuau...........   8       0    seq
    ....................................................cagcauuguacagggcu............   1       0    seq
    ....................................................cagcauuguacagggcuau..........   3       0    seq
    ....................................................cagcauuguacagggcuauc.........   3       0    seq
    ....................................................cagcauuguacagggcuauca........   1       0    seq
    ....................................................cagcauuguacagggcuauGaaa......   3       1    seq
```
